# Blood Pressure Variability and Low-Grade Inflammation in Pediatric Patients with Primary Hypertension

**DOI:** 10.3390/jcm14165737

**Published:** 2025-08-13

**Authors:** Katarzyna Dziedzic-Jankowska, Michał Szyszka, Adam Bujanowicz, Anna Stelmaszczyk-Emmel, Piotr Skrzypczyk

**Affiliations:** 1 Department of Pediatrics and Nephrology, Medical University of Warsaw, 02-091 Warsaw, Poland; katarzyna_dziedzic11@wp.pl; 2 Department of Pediatrics and Nephrology, Doctoral School, Medical University of Warsaw, 02-091 Warsaw, Poland; mszyszka@wum.edu.pl (M.S.); adam.bujanowicz@wum.edu.pl (A.B.); 3 Department of Laboratory Diagnostics and Clinical Immunology of Developmental Age, Medical University of Warsaw, 02-091 Warsaw, Poland; anna.stelmaszczyk-emmel@wum.edu.pl

**Keywords:** primary hypertension, children, blood pressure variability, blood pressure dipping, low-grade inflammation, monocyte-to-neutrophil ratio

## Abstract

**Background/Objectives**: Increased blood pressure variability (BPV) was found in adults with primary (essential) hypertension (PH) and is associated with increased cardiovascular risk. Our study aimed to analyze the relation between BPV and low-grade inflammation in children with primary hypertension. **Methods**: In 56 treatment-naive pediatric patients with PH (15.1 ± 2.1 years) and 30 healthy children (14.9 ± 1.4 years), we evaluated BPV: BP dipping, standard deviation (SD) of ambulatory blood pressure measurements (ABPMs), pulse pressure (PP)/systolic blood pressure ratio (24 h PP/SBP), rate–pressure index (24 h RPI), 24-h weighted BPV (24 h WSBPV, 24 h WDBV, 24 h WMAPV), coefficient of variation (24 h CoVSBP, 24 h CoVDBP, 24 h CoVMAP), ambulatory arterial stiffness index (AASI), and morning BP surge. We also analyzed indices of subclinical inflammation (markers derived from complete blood count, high-sensitivity C-reactive protein (CRP), interleukin 18), and office and ambulatory BP. **Results**: Patients with PH had significantly higher hsCRP, neutrophils, monocytes, and platelets, neutrophil-to-lymphocyte (NLR), platelet-to-mean platelet volume (PMPVR), and lower monocyte-to-neutrophil (MNR) ratios, and higher BPV: 24 h ABPM SBP SD, 24 h ABPM MAP SD, 24 h RPI, 24 h WSBPV, 24 h WDBV, 24 h WMAPV, and 24 h CoVSBP. Low-grade inflammation markers correlated with BPV indices in both groups. In multivariate analysis, MNR predicted 24 h ABPM MAP SD (beta = 0.290, 95CI: 0.029–0.551), 24 h RPI (beta = −0.348, 95CI: −0.587–−0.108), and 24 h WDBPV (beta = 0.286, 95CI: 0.032–0.540); monocyte count—24 h RPI (beta = 0.281, 95CI: 0.041–0.521), and hsCRP—24 h WDBV (beta = 0.310, 95CI: 0.055–0.564). ROC analysis revealed a good diagnostic profile for lymphocyte count as a positive determinant of non-dipping status in PH children (cut-off point 2.59 [×10^3^/µL]). **Conclusions**: BPV is higher in children with PH compared to healthy peers and is associated with low-grade inflammation. MNR may be the most helpful indicator of BPV, whereas high lymphocyte count predicts the best non-dipping status in these patients.

## 1. Introduction

Arterial hypertension (AH) is one of the key preventable causes of death, with a worldwide prevalence estimated for approximately 30% of adults [1,2]. According to a recently published meta-analysis, the prevalence of AH in the pediatric population is approximately 4.0% [3]. Of the many etiologies of hypertension in developmental age, primary hypertension (PH), as in adults, is the most important single cause of elevated blood pressure among adolescents [4]. While the pathogenesis of PH is not fully understood and is certainly multifactorial, several concepts have been proposed to explain the rise in blood pressure, often as early as the first decade of life. It is hypothesized that genetic factors, epigenetic modifications (e.g., microRNAs), intestinal microbiota abnormalities, increased sympathetic activity, the renin–angiotensin–aldosterone system, and low-grade (i.e., subclinical) inflammation may all be involved in the pathogenesis of PH [5].

Performing 24 h ambulatory blood pressure monitoring (ABPM) has become the standard for diagnosing and monitoring pediatric patients with hypertension. International recommendations suggest that ABPM is a valuable tool for confirming hypertension, assessing the degree of arterial hypertension (AH) control, and ruling out white coat hypertension (WCH) [6,7]. It is notable that ABPM also allows for the assessment of several parameters above standard mean pressures, such as pressure variability parameters (BPV) [8].

Blood pressure variability (BPV) is considered nowadays as a prognostic factor increasing the risk of cardiovascular sequelae [9]. Variation occurs due to several factors, including those emanating from the person, environment, atmosphere, and time of day. BPV has been noted in both normotensive and hypertensive subjects. BPV can be physiological, enabling an individual to cope with the stress of daily life, or pathological, serving as a harbinger of disease [8,10,11]. BPV can be assessed based on a vast number of indicators, like standard deviations of blood pressure measurements or nighttime blood pressure dipping, for many of which prognostic significance has been demonstrated in adults [8]. However, their role in pediatrics remains unclear. It is challenging to determine which of the numerous indicators would be most valuable in assessing cardiovascular risk and prognosis in this patient group [12].

As mentioned above, the activation of low-grade inflammation appears to be one of the pillars of PH pathogenesis. In numerous papers, including those by our team, higher inflammatory parameters were found in patients with PH compared to healthy peers [13,14,15,16]. A meta-analysis of 13 pediatric studies published by our group indicated that high-sensitivity C-reactive protein (hs-CRP) and adhesion molecules concentrations, neutrophil, monocyte and platelet counts, and the neutrophil-to-lymphocyte ratio were higher and the lymphocyte-to-monocyte ratio was lower in children with PH [17]. Only a few adult [18,19,20] and a single pediatric study [21] analyze the associations between subclinical inflammation and blood pressure variability. It should be emphasized that the previous manuscripts of our team [13,14,15,16], as well as the meta-analysis already cited [17], showed differences in inflammatory markers between children with PH and normotensive children, but did not assess differences in the extent of variability and the relationship between blood pressure variability and inflammation. It cannot be ruled out that there is a reciprocal relationship between low-grade inflammation and BPV, which ultimately results in hypertension-mediated organ damage (HMOD) and increased cardiovascular risk.

Hence, the objectives of our study are as follows:

1. To compare different BPV indices between children with untreated PH and healthy children.
2. To determine the relationship between BPV indices and inflammatory parameters in these groups of patients.

## 2. Materials and Methods

**Figure 1:**
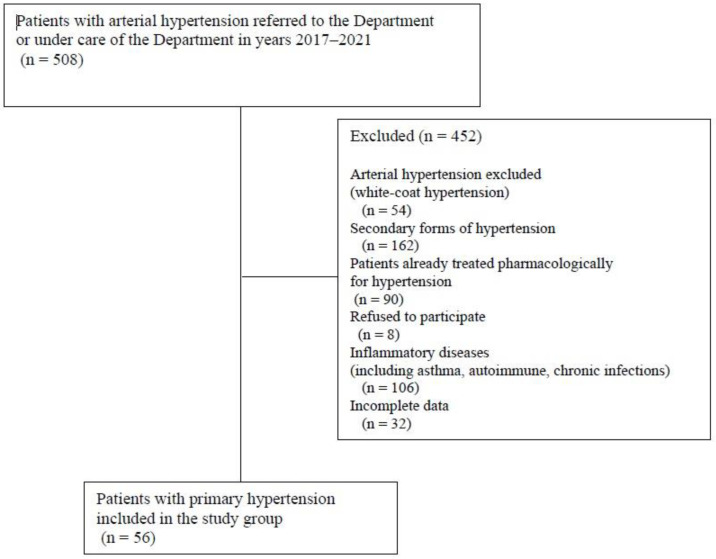
Flowchart of children with primary hypertension included in study group.

Our retrospective analysis included all children and adolescents hospitalized in our tertiary pediatric nephrology center with suspected AH between 2017 and 2021. The inclusion criterion for the PH group was a diagnosis of AH by the European 2016 guidelines [17] and finally confirmed in ABPM. The control group (CG) comprised 30 age- and sex-matched healthy children. The exclusion criteria for all the participants were acute and chronic inflammatory conditions (including, among others, autoimmune, rheumatic diseases, inflammatory bowel syndrome), allergic/atopic diseases, chronic kidney disease, and severe heart diseases (heart defects or heart failure). In addition, we excluded all the patients with secondary forms of hypertension and those receiving pharmacological antihypertensive treatment. In all study participants, potential sources of inflammation that could have influenced the results were analyzed based on medical records (physical examination and history)—such patients were excluded from the analysis. Figure 1 shows the patients’ flowcharts.

The researchers received approval from the local bioethics committee (approval No. KB/58/2016, 15 March 2016, amendment No. KB/53/A2023, 12 June 2023). The study was conducted by following the highest ethical standards and the Helsinki Declaration. All participants and their legal representatives signed informed consent forms before entering the study.

In all the analyzed children, we evaluated age, sex, duration of PH, pregnancy duration, and birthweight. Height, weight, and body mass index (BMI) were presented as direct values and Z-scores [18]. We defined overweight and obesity as BMI ≥ 85th and <95th percentile, and ≥95th percentile.

We measured peripheral office blood pressure by Welch Allyn VSM Patient Monitor 300 (Welch Allyn Inc., Skaneateles Falls, NY, USA) [mmHg] [17]. Blood pressure was expressed as [mmHg] and as Z-scores [19]. We calculated pulse pressure (PP) using the following definition: PP = SBP − DBP [mm Hg].

In all the children, we used a Suntech Oscar 2 monitor (SunTech Medical, Inc., Morrisville, NC, USA) to perform ABPM. We analyzed [20] systolic, diastolic, and mean blood pressure during 24 h (SBP, DBP, MAP, 24 h) [mm Hg], Z-scores [20], systolic and diastolic 24-h blood pressure loads (BPL) [%], and 24-h pulse pressure (PP).

Based on the available literature, we calculated the following blood pressure variability indices [8,22]:

- BP variability—the standard deviation (SD) of systolic, diastolic, and mean blood pressure within 24 h (24 h SBPV, 24 h DBPV, 24 h MAPV).
- Systolic and diastolic blood pressure dipping (SBP DIP, DBP DIP)—the difference between daytime and nighttime blood pressure expressed in percentages of daytime blood pressure [%].
- Morning blood pressure surge was defined as the mean BP in the first 2 h after wake-up minus the mean BP 2 h before wake-up [mm Hg].
- PP/SBP ratio—PP/SBP during 24 h (24 h PP/SBP).
- Rate–pressure product (index)—heart rate multiplied by systolic blood pressure during 24 h (24 h RPI) [bpm·mm Hg].
- The variation in 24-h weighted blood pressure was defined as the mean of daytime and nighttime BP adjusted for the day and night period (the mean of the daytime and nighttime SDs, weighted for the duration of the daytime and nighttime periods) (24 h WSBPV, 24 h WDBV, 24 h WMAPV).
- Coefficient of variation (CoV) was defined as blood pressure standard deviation/divided by mean BP expressed in percentiles during 24 h (24 h CoVSBP, 24 h CoVDBP, 24 h CoVMAP) [%].
- Ambulatory arterial stiffness index (AASI) was defined as one minus the regression slope of DBP over SBP 24 h ABPM values [23].

We evaluated subclinical (low-grade) inflammation using serum markers and complete blood count (CBC) markers. We collected blood in the morning after a 12 h fasting period in a euvolemic state per local protocol. All patients were recommended a normal sodium diet for at least seven days before admission. We analyzed peripheral blood morphology using a Sysmex XN1000 (Sysmex Corporation, Kobe, Japan) hematologic analyzer. We evaluated neutrophil, lymphocyte and platelet counts, mean platelet volume (fL), and the following indices (ratios): neutrophil-to-lymphocyte, platelet-to-lymphocyte, monocyte-to-lymphocyte, monocyte-to-neutrophil, and platelet-to-mean platelet volume (10^12^/fL). The ELISA kits were used to measure serum concentrations of hs-CRP (DRG^®^ CRP, DRG International Inc., Springfield, NJ, USA) and interleukin-18 (IL-18) (Human IL-18 ELISA Kit, Thermofisher Scientific, Vienna, Austria).

The remaining biochemical parameters were evaluated using standard local laboratory methods (dry chemistry (VITROS 5600, Ortho Clinical Diagnostics, Raritan, NJ, USA)). Abnormal albuminuria was defined as an albumin-to-creatinine ratio equal to or exceeding 30 mg/g [17]. We defined hyperuricemia as a serum uric acid concentration exceeding 5.5 mg/dL [23].

Because of the retrospective nature of the study, three sets of data were incomplete: urinary albumin excretion (*n* = 85), duration of pregnancy (*n* = 72), and birth weight (*n* = 53).

The participants’ data was analyzed with Statistica 13.0 PL (TIBCO Software Inc., Palo Alto, CA, USA). The heatmaps were prepared using Python 3.12 (Python Software Foundation, Wilmington, DE, USA). Based on the literature data, we estimated the sample at 30 children in each group (statistical power of 0.8, *p* = 0.05, effect size of 0.50). We presented the results as numbers, mean values with standard deviation (SD), and interquartile range (IQR). We implemented the statistical tests (depending upon the variables’ distribution): the Shapiro–Wilk test (test for normality of data sets), student *t*-test, the U Mann–Whitney test, Pearson’s linear correlation, Spearman’s rank correlation, chi-square test, and receiver operating characteristic (ROC) analysis. A general regression model (GRM) was created to perform a multivariate analysis. Parameters that correlated with BPV with *p* < 0.10 were included in the final GRM analysis. A *p* < 0.05 was considered statistically significant.

## 3. Results

We provide the basic parameters of the studied children in Table 1. Age and sex were comparable in both groups. BMI was significantly higher in children with PH compared to their healthy peers. There were 19 (33.9%) overweight and 17 (30.4%) obese children in the PH group. The groups did not differ in serum urea and creatinine, but uric acid was significantly higher in patients with PH. Thirty-four (50.6%) patients with PH had hyperuricemia. Regarding lipid parameters, HDL-cholesterol concentrations were significantly lower, and triglyceride concentrations were significantly higher in patients with PH. As for markers of subclinical inflammation, the groups differed significantly in hsCRP concentration, neutrophil, monocyte, and platelet counts, as well as in the following indices: NLR, MNR, and PMPVR. There was no difference in interleukin 18 concentration.

Table 2 shows the blood pressure values and variability in both groups. All blood pressure indices were significantly higher in the study group compared to the healthy peers. Notably, the groups did not differ in their heart rates. We found numerous differences in indices of blood pressure variability between the groups. Patients with primary hypertension had significantly higher 24 h ABPM SBP and MAP standard deviations, 24 h rate–pressure index, 24 h weighted SBP, DBP, and MAP variations, and a lower 24 h coefficient of SBP variation. Non-dipping pattern of BP rhythm was found in 17/56 (30.4%) patients with PH, and in 7/30 (23.3%) healthy children (*p* = 0.489).

**Figure 2:**
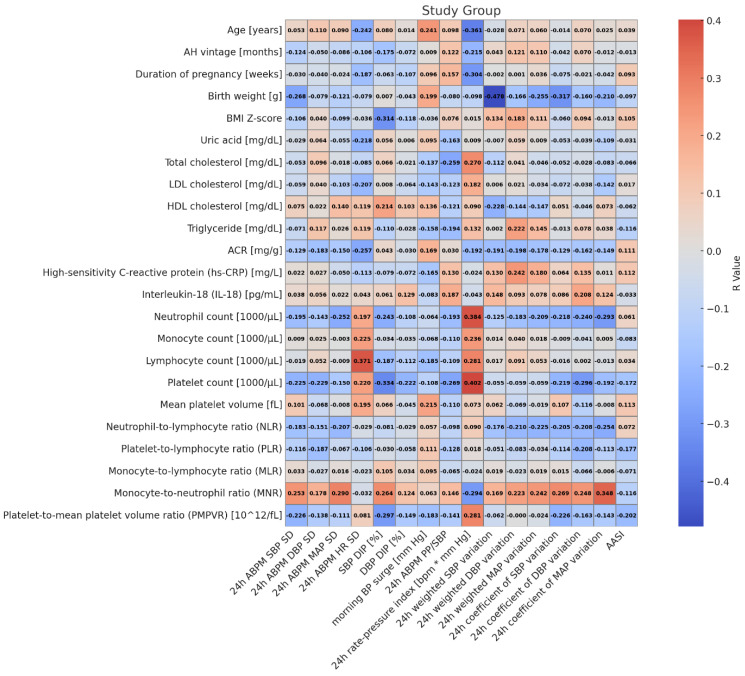
Heatmap of correlations between markers of blood pressure variability and markers of inflammation in patients with hypertension.

**Figure 3:**
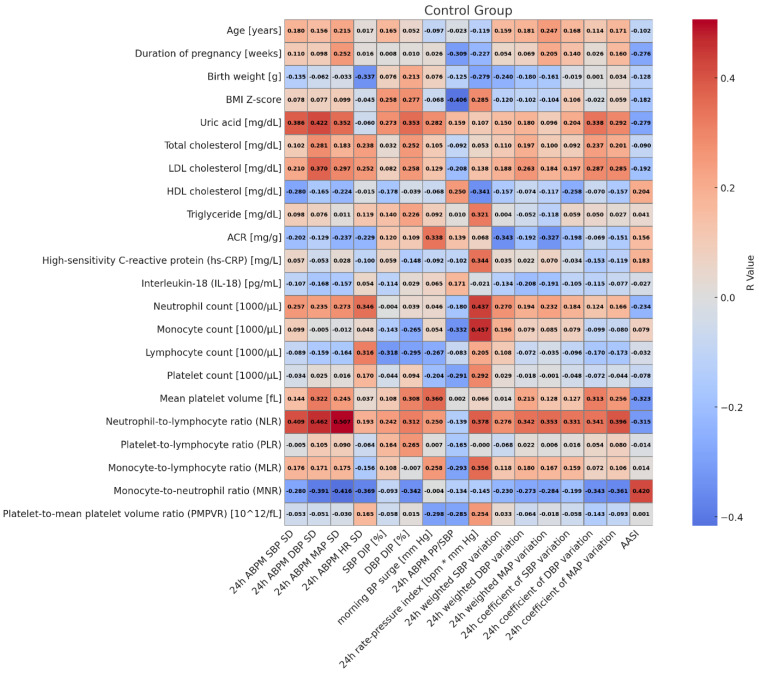
Heatmap of correlations between markers of blood pressure variability and markers of inflammation in healthy children.

Figure 2 and Figure 3 show the correlations between indices of blood pressure variability and clinical and biochemical markers, and markers of subclinical inflammation in patients with primary hypertension and healthy children. In hypertensive patients, MAP 24 h SD correlated positively with MNR (r = 0.290, *p* = 0.030); HR 24 h SD correlated with lymphocyte count (r = 0.372, *p* = 0.005), diastolic blood pressure dipping correlated with BMI Z-score (r = −0.314, *p* = 0.019), platelet count (r = −0.334, *p* = 0.012), MNR (r = 0.264, *p* = 0.0049), and PMPVR (r = −0.298, *p* = 0.026); 24 h PP/SBP correlated with platelet count (r = −0.269, *p* = 0.045); 24 rate–pressure index correlated with age (r = −0.361, *p* = 0.006), neutrophil count (r = 0.384, *p* = 0.004), lymphocyte count (r = 0.282, *p* = 0.036), platelet count (r = 0.402, *p* = 0.002), MNR (r = −0.294, *p* = 0.028), PMPVR (r = 0.282, *p* = 0.036), and total cholesterol (r = 0.270, *p* = 0.045); 24 h weighted SBP variation correlated with birth weight (r = −0.478, *p* = 0.009); 24 h coefficient of SBP variation correlated with MNR (r = 0.269, *p* = 0.045); 24 h coefficient of DBP variation correlated with platelet count (r = −0.296, *p* = 0.027); and 24 h coefficient of MAP variation correlated with neutrophils (r = −0.293, *p* = 0.028) and MNR (r = 0.348, *p* = 0.009).

In healthy children, SBP 24 h SD correlated with NLR (r = 0.409, *p* = 0.025) and uric acid (r = 0.386, *p* = 0.035); DBP 24 h SD correlated with NLR (r = 0.462, *p* = 0.010), MNR (r = −0.391, *p* = 0.033), uric acid (r = 0.422, *p* = 0.020), LDL-cholesterol (r = 0.37, *p* = 0.044); MAP 24 h SD correlated with NLR (r = 0.507, *p* = 0.004) and MNR (r = −0.416, *p* = 0.022); SBP dipping correlated with body weight (r = 0.361, *p* = 0.050); AASI correlated with MNR (r = 0.420, *p* = 0.021); 24 h PP/SBP correlated with body weight Z-score (r = −0.365, *p* = 0.047), BMI (r = −0.382, *p* = 0.037), and BMI Z-score (r = −0.406, *p* = 0.026); 24 h rate pressure index correlated with neutrophil count (r = 0.437, *p* = 0.016), and monocyte count (r = 0.457, *p* = 0.011), NLR (r = 0.378, *p* = 0.039); and 24 h coefficient of MAP variation correlated with NLR (r = 0.396, *p* = 0.030).

**Figure 4:**
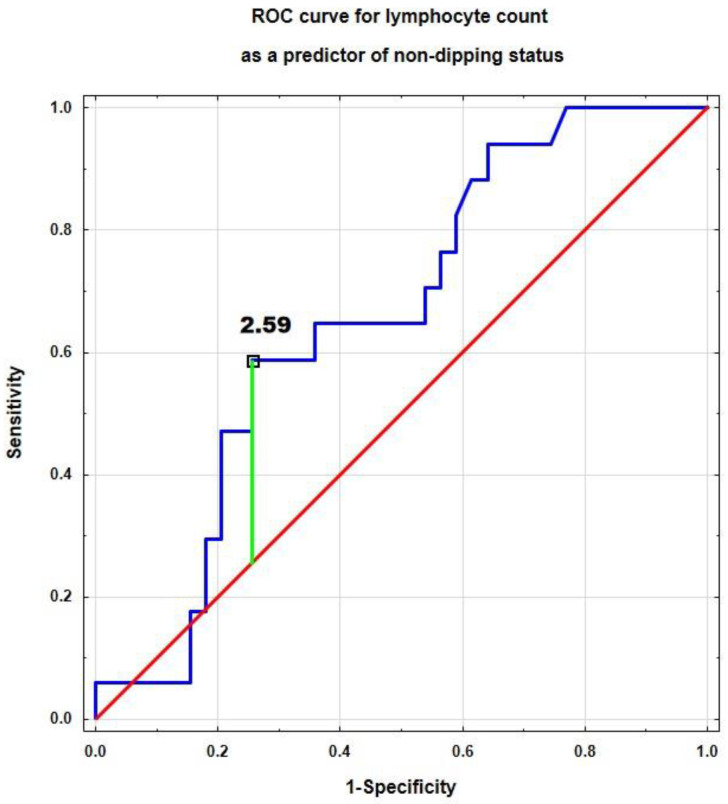
Receiver operating characteristic (ROC) analysis of lymphocyte count as predictor of non-dipping status in patients with primary hypertension.

We performed a ROC analysis to determine the immunological determinants of non-dipping status in children with primary hypertension (Table 3). Only the lymphocyte count was a significant predictor of non-dipping status with a good diagnostic profile (area under the curve, sensitivity, and specificity). The cut-off point for lymphocyte count was 2.59 [×10^3^/µL] (Figure 4).

In multivariate analysis, the monocyte/neutrophil ratio was a significant immunological predictor of the following indices of blood pressure variability: 24 h ABPM MAP SD, 24 h rate–pressure index, and 24 h weighted DBP variation. Additionally, monocyte count was a predictor of 24 h rate–pressure index, and hsCRP predicted 24 h weighted DBP variation (Table 4).

## 4. Discussion

In our single-center cross-sectional study, we have shown that children with primary hypertension, compared to healthy peers, were characterized by significantly higher markers of subclinical inflammation, i.e., hsCRP, neutrophil, monocyte, and platelet counts, and the following indices: neutrophil-to-lymphocyte ratio and platelet-to-mean platelet volume ratio; conversely, the monocyte-to-neutrophil ratio was significantly lower in hypertensive children. Children with PH were characterized by significantly higher values of blood pressure variability indices, 24 h ABPM SBP SD, 24 h ABPM MAP SD, 24 h rate–pressure index, 24 h weighted SBP, DBP, and MAP variations, and lower 24 h coefficient of SBP variations. In children with PH, we found numerous correlations between markers of subclinical inflammation and markers of blood pressure variability, such as between MAP 24 h SD and MNR, or between the 24 h rate–pressure index and neutrophil, lymphocyte, and platelet counts. In ROC analysis, lymphocyte count was a significant predictor of non-dipping status. Multivariate analysis revealed that MNR was the most common independent determinant of variation indices in children with PH.

Measuring office blood pressure values remains the gold standard for the diagnosis and monitoring of hypertensive patients. Recent studies have shown that the evaluation of BP variability is important in assessing patients’ cardiovascular risk [8,24,25]. Increased BPV is positively correlated with risk of hypertension-mediated organ damage, negative sequelae, and all-cause mortality [11,26]. A meta-analysis of 41 papers showed that long-term, mid-term, and short-term variability are associated with cardiovascular risk above the effect of the mean blood pressure [27]. Controlling BPV in addition to decreasing BP may be another goal for the treatment of patients with arterial hypertension [11]. Blood pressure variability was found to be correlated with blood pressure values. Thus, indices of BPV are higher in patients with arterial hypertension compared to normotensives [28]. It was also revealed that a reduction in blood pressure proportionally decreases blood pressure variability [10].

In our cohort, numerous indices of blood pressure variability were significantly higher in hypertensive children compared to healthy ones. This is in line with adult studies where hypertensive patients were also characterized by increased BP variability [29]. In children, similarly to adults, increased blood pressure variability was found to be a risk factor for hypertension-mediated organ damage. A recently published American SHIP AHOY study revealed that increased BP variability is a risk factor for higher left ventricular mass index and diastolic dysfunction [30]. Similar results were shown by authors from Egypt [31], China [32], and Canada [33]. High blood pressure variability was also found to increase arterial stiffness in children [34]. The proposed cause for increased blood pressure variability in patients with arterial hypertension is primarily increased activity of the sympathetic nervous system. Additionally, other contributing factors are mentioned, e.g., increased activity of the renin–angiotensin–aldosterone (RAA) system, decreased arterial and cardiopulmonary reflexes, and increased arterial stiffness [10,12]. The mechanism linking variability to organ damage and cardiovascular risk is also unclear. It has been postulated that high variability is a marker of sympathetic and renin–angiotensin–aldosterone (RAA) system activation, which in turn negatively affects target organs. Additionally, variability may be considered a specific biomarker indicating pre-existing organ damage (vascular stiffness). In addition, rapidly changing the load and hemodynamic stress exerted on arterioles can lead to the development of arteriosclerosis and further organ damage [27,35,36].

In our cohort, inflammatory markers were higher in children with PH compared to those in the healthy group. PH is considered to be a complex condition that runs the gamut of immune disorders, metabolic disorders, and early biological aging, including early vascular aging [5]. Our results are consistent with those of many studies in adults and children, including a recent meta-analysis published by our team [17]. In one of our previous manuscripts, we also compared the severity of low-grade inflammation in patients with white coat hypertension and found that this group of patients had a similar level of inflammation compared to patients with PH [13].

The first reports on the relationship between arterial hypertension and inflammation date back to the 1960s [37]. Nevertheless, it was only the discoveries of the last 25 years (including the research of a Polish-origin researcher, Tomasz Guzik) that resulted in a deeper understanding of the immunological processes involved in patients with primary hypertension [38]. The current hypothesis is that factors such as a slight increase in blood pressure and shear stress damage endothelial cells, resulting in the release of neoantigens and stimulation of the immune system. The NLRP3 inflammasome, sympathetic overdrive, and a high-sodium diet are additional triggers of immune activation. These factors shift the balance of helper lymphocytes (Th) toward interleukin-17-producing lymphocytes (Th17), while simultaneously impairing the function of regulatory lymphocytes (Tregs) [39,40,41]. Interleukin 17, in the absence of the inhibitory effect of Tregs, causes sodium retention and vasoconstriction, which results in a further increase in blood pressure and perpetuation of hypertension [42]. Interleukin 17, interferon-γ, and tumor necrosis factor-α are considered pro-hypertensive cytokines, whereas interleukin-10 may play a protective role [38].

As can be seen, even in this simplified model, the relationship between inflammation and blood pressure is complex, and in clinical practice, it is not easy to pinpoint which came first. Add to this the influence of obesity, especially abdominal obesity, which is itself associated with elevated inflammatory markers, and the situation becomes more complex. Adipose tissue, especially visceral adipose tissue, is a source of many pro-inflammatory and blood pressure-raising substances [43]. It should be noted that overweight or obesity is present in more than half of children and adolescents with primary hypertension [44].

Our analysis revealed numerous positive correlations between BP variability indices and inflammatory markers. Similar results were already found in adult studies for interleukin 6 [18,36], CRP [45,46], and VCAM-1 [46]. Interestingly, Greek authors reported significantly higher indices of blood pressure variability compared to healthy controls in normotensive patients with psoriasis, an immune-mediated chronic inflammatory disease [47]. Additionally, in patients with systemic lupus erythematosus, blood pressure variability indices have been shown to correlate positively with disease activity [48,49].

Multivariate analysis revealed that CRP concentration, monocyte count, and MNR were the strongest predictors of blood pressure variability in our cohort of hypertensive subjects. Elevated CRP is a well-known predictor of cardiovascular events and correlates positively with the risk of all-cause and cardiovascular death in adults [50]. Elevated CRP has also been described by other authors in children with primary hypertension [17]. Our study aligns with existing adult data, which suggests a positive association between CRP concentration and blood pressure variability indices [45,46].

Monocyte count and the monocyte/neutrophil index are recently studied markers of subclinical inflammation [51,52,53,54]. Their importance may be the consequence of the potential role of monocytes in raising blood pressure [55,56], the development of left ventricular hypertrophy [57], and finally in the formation of atherosclerotic lesions (as lipid-packed macrophages—foam cells) [58]. In our previous study, we demonstrated that MNR was the most significant immunological predictor of left ventricular hypertrophy in untreated children with primary hypertension [59]. The results of this study provide further evidence of the utility of MNR. There is no doubt that the usefulness of this simple and everywhere available index should be evaluated in prospective and interventional studies.

Musiał et al. [21] revealed numerous correlations between CBC-derived markers of inflammation and blood pressure (BP) variability in pediatric patients with arterial hypertension (the authors analyzed primary and secondary hypertension separately). Notably, many of these correlations (NLR, PLR) were negative, and the authors analyzed only one index of BPV—the standard deviation of ABPMs [21]. In the study by Musiał, LMR was the strongest predictor of blood pressure (BP) variability, and this association was observed for both primary and secondary hypertension, as well as for dippers and non-dippers [21]. It is difficult to clearly explain the differences in results between our study and the above study. We used the same apparatus for ABPM, i.e., Suntech Oscar 2 (SunTech Medical, Inc., Morrisville, NC, USA), and the age of the children studied and the size of the groups were also comparable. Many of the correlations shown in univariate analysis may be apparent but do not account for additional confounding factors. Hence, in our study, we conducted multivariate analysis, which highlighted the importance of the MNR index as a predictor of variability.

It is important to highlight the differences between this study and previous studies by our team and other teams. The topic of differences in inflammatory parameters has already been the subject of many papers, including the meta-analysis we published [17]. Nonetheless, this is the first pediatric paper, except for the cited study by Musial et al. [21], that analyzes the parameters of pressure variability and the relationship between variability and inflammation in a pediatric population with PH. This is what we believe distinguishes our results as innovative and original.

The exact pathophysiological link between BP variability and inflammation has not been elucidated. Interesting data have been provided by studies on an animal model of blood pressure (BP) variability, specifically sinoatrial denervation (SAD) in rats. In these animals, blood pressure (BP) variability and inflammatory markers are elevated, and increased organ damage is observed [60]. Interestingly, sinoatrial denervation in spontaneously hypertensive rats (SHRs) increased the hypertrophy and fibrosis of the myocardium and impaired systolic function without increasing blood pressure values. SAD raised the expression of transforming growth factor-beta and chemoattractant protein-1, and induced infiltration of myocardium with macrophages [61]. Additionally, in these rats, anti-inflammatory treatment (indomethacin and vitamin E) decreased myocardial and vascular fibrosis and the infiltration of inflammatory cells, and alleviated renal damage [62].

The non-dipping phenomenon is one of the most studied variabilities in both the child and adult populations. A non-dipping pattern is independently associated with increased cardiovascular risk in adults [63] and increased risk for HMOD in children [64]. In our recent study, we found that a disturbed circadian BP profile was common in children with primary hypertension; thus, it should not be considered a marker of secondary hypertension. Non-dipping was associated with higher left ventricular mass, and extreme dipping was associated with arterial stiffness [65]. In this study, we revealed no differences between patients with PH and healthy patients in terms of SBP or DBP dipping. In our hypertensive patients, systolic blood pressure dipping correlated negatively with BMI and inflammatory indices, negatively with platelets, and positively with MNR and PMPVR. In the control group, diastolic blood pressure dipping correlated positively with MNR. In a ROC analysis, lymphocyte count was the only significant predictor of non-dipping pattern. Similarly, in numerous adult studies, a non-dipping pattern was associated with elevated inflammatory markers—NLR [58], PLR [58], MLR [58], MPV [59], soluble intercellular adhesion molecule-1 [60], hsCRP [59,61], and the monocyte/cholesterol ratio [66]. Hence, our results confirm the relationship between inflammation and the non-dipping phenomenon, which in turn may increase the risk of HMOD and cardiovascular complications.

In a Turkish adult study, interleukin-18 was not only higher in patients with newly diagnosed hypertension compared to the control group, but its concentration was strictly related to the non-dipping pattern [67]. Interleukin-18 is a pro-inflammatory cytokine produced mainly by monocytes and macrophages and boosts interferon γ production by T and NK lymphocytes. Both interleukin-18 and its receptors were found in endothelium and vascular smooth muscle cells [68]. In our pediatric cohort, there were no differences in concentrations of this cytokine between patients with PH and the control group. Also, no correlations with variability or dipping were revealed. This difference may be due to less advanced vascular changes (children with primary hypertension do not have atherosclerosis, and arterial changes are adaptive and are described as arteriosclerosis) [69].

It is worth noting the different relationships between inflammatory markers and markers of variability in the two groups. Thus, for example, for the already discussed MNR, the correlations in the study group were primarily positive, while those in the control group were mostly negative. Considering the difference in MNR values between the groups, this may indicate significant differences in cardiovascular regulation in healthy and PH children. It has been shown that children with PH differ in many biological aspects from their healthy peers. Among other things, Litwin’s group showed differences in body composition (increased fatty tissue) [70] and a more advanced vascular [71] and skeletal age [72] in adolescents with PH.

The ambulatory arterial stiffness index is an indirect marker of vascular stiffness [23]. Although a recently published meta-analysis showed that in adults, higher AASI is a risk factor of all-cause and cardiovascular death, stroke, and major adverse cardiovascular events [73], its usefulness in children is questioned [74]. In our study, there were no differences in AASI between the groups. Moreover, only in the group of healthy children was there a significant positive correlation between AASI and MNR. The measurement of aortic pulse wave velocity (using applanation tonometry or oscillometry) remains the gold standard of evaluation of arterial stiffness in children. In one of our previous manuscripts, we found that NLR might be a promising biomarker of local and central arterial stiffness in adolescents with PH [16].

The strengths of our study are as follows: the evaluation of a homogeneous group of patients with untreated primary hypertension, with no factors that could influence inflammation status. The group size was sufficient to draw reliable conclusions. Additionally, it is worth noting that a significant number of indicators of pressure variability and numerous markers of subclinical inflammation were examined. The undoubted limitations of this study are its cross-sectional nature and lack of evaluation of organ changes. Although the ABPM device used has passed validation, it has only been validated in the adult patient population, and ESH recommends its use in these patients. The group was very homogeneous; therefore, it is unclear whether one can generalize our results to other populations, such as young adults or patients from different ethnic groups. Finally, we cannot exclude other hidden sources of inflammation in our patients, such as those who do not undergo ear, nose, and throat or dental examinations [75].

## 5. Conclusions

Our study revealed that children with primary hypertension have higher markers of subclinical inflammation and also significantly higher blood pressure variability. We have also found multiple correlations between these two sets of data. Multivariate analysis suggests that the monocyte-to-neutrophil ratio might be the most useful biomarker of blood pressure variability in hypertensive children. Conversely, lymphocyte count may be a significant predictor of non-dipping status.

## Figures and Tables

**Table 1:** Clinical, biochemical, and inflammatory parameters in studied children.

Parameter	Primary Hypertension	Control Group	*p*
Number of patients (*n*)	56	30	-
Age [years]	15.1 ± 2.3 (13.9–16.8)	14.9 ± 1.4 (13.8–15.9)	0.159
Sex [M/F]	40/16	19/11	0.472
Duration of PH [months]	12.57 ± 0.86 (3.0–17.0)	-	-
Duration of pregnancy [weeks]	39.3 ± 2.0 (40.0–40.0)	39.5 ± 1.5 (40.0–40.0)	0.564
Birth weight [g]	3201.1 ± 634.9 (2920–3600)	3435.4 ± 416.3 (3050–3790)	0.133
BMI Z-score	1.47 ± 0.86 (0.92–2.16)	0.13 ± 0.72 (−0.30–0.75)	<0.001
Serum creatinine [mg/dL]	0.8 ± 0.2 (0.6–0.9)	0.7 ± 0.1 (0.6–0.8)	0.129
Serum urea [mg/dL]	26.4 ± 5.7 (22.5–30)	25.1 ± 5.5 (22–28)	0.328
Serum uric acid [mg/dL]	6.1 ± 1.5 (5.2–6.9)	5.1 ± 1.1 (4–5.7)	0.002
Serum total cholesterol [mg/dL]	163.9 ± 26.0 (145.5–179)	157.2 ± 30.7 (133–182)	0.233
Serum LDL-cholesterol [mg/dL]	90.4 ± 24.3 (69.5–105.7)	86.2 ± 27.2 (64.6–109)	0.447
Serum HDL-cholesterol [mg/dL]	50.7 ± 12.9 (43–58.5)	57.8 ± 11.1 (50–65)	0.013
Serum triglyceride [mg/dL]	113.7 ± 56.9 (65.5–144)	65.7 ± 25.9 (50–78)	<0.001
Urinary ACR [mg/g]	17.5 ± 47.2 (5.0–11.4)	9.8 ± 8.7 (4–11.8)	0.566
hsCRP [mg/L]	4.9 ± 5.2 (1.5–7.3)	2.8 ± 5.4 (0.5–1.9)	<0.001
IL-18 [pg/mL]	81.8 ± 76.7 (21.6–130.4)	92.5 ± 58.1 (43.7–117.5)	0.201
Neutrophil count [×10^3^/µL]	3.89 ± 1.44 (2.88–4.80)	2.63 ± 0.96 (1.94–3.16)	<0.001
Monocyte count [×10^3^/µL]	0.56 ± 0.17 (0.45–0.65)	0.46 ± 0.14 (0.34–0.53)	0.003
Lymphocyte count [×10^3^/µL]	2.44 ± 0.88 (1.90–2.71)	2.09 ± 0.49 (1.70–2.38)	0.060
Platelet count [×10^3^/µL]	263.7 ± 55.3 (227.0–297.0)	239.2 ± 52.6 (215–258)	0.037
Mean platelet volume (MPV) [fL]	10.44 ± 1.34 (9.70–11.35)	10.92 ± 0.73 (10.20–11.50)	0.069
Neutrophil-to-lymphocyte ratio (NLR)	1.73 ± 0.88 (1.22–2.0)	1.27 ± 0.41 (0.92–1.55)	0.005
Platelet-to-lymphocyte ratio (PLR)	117.15 ± 34.25 (88.75–137.87)	118.59 ± 32.35 (98.04–145.68)	0.851
Monocyte-to-lymphocyte ratio (MLR)	0.25 ± 0.08 (0.19–0.30)	0.22 ± 0.06 (0.18–0.26)	0.173
Monocyte-to-neutrophil ratio (MNR)	0.16 ± 0.06 (0.11–0.19)	0.19 ± 0.05 (0.14–0.22)	0.010
Platelet-to-MPV ratio (PMPVR) (10^12^/fL)	25.81 ± 6.76 (20.85–30.09)	22.02 ± 5.11 (19.8–23.45)	0.010

M—male; F—female; PH—primary hypertension; BMI—body mass index; LDL—low-density lipoprotein; HDL—high-density lipoprotein; ACR—urinary albumin-creatinine ratio; hsCRP—high-sensitivity C-reactive protein; IL-18—interleukin-18.

**Table 2:** Blood pressure and blood pressure variability in the studied children.

Parameter	Primary Hypertension	Control Group	*p*
Office SBP [mm Hg]	141.7 ± 10.2 (135–148)	113.5 ± 7.6 (109–119)	<0.001
Office SBP Z-score	2.26 ± 0.90 (1.81–2.94)	−0.15 ± 0.81 (−0.64–0.44)	<0.001
Office DBP [mm Hg]	83.1 ± 10.2 (76–91)	65.1 ± 5.8 (60–69)	<0.001
Office DBP Z-score	2.39 ± 1.39 (1.36–3.47)	−0.01 ± 0.81 (−0.66–0.55)	<0.001
24 h ABPM SBP [mm Hg]	134.6 ± 5.3 (131–138.5)	113.4 ± 5.9 (110–118)	<0.001
24 h ABPM SBP Z-score	2.34 ± 0.86 (1.73–2.76)	−0.31 ± 0.68 (−0.85–0.25)	<0.001
24 h ABPM DBP [mm Hg]	72.2 ± 7.1 (69–77)	62.7 ± 3.8 (60–66)	<0.001
24 h ABPM DBP Z-score	0.77 ± 1.27 (0.20–1.54)	−0.91 ± 0.74 (−1.37–−0.42)	<0.001
24 h ABPM MAP [mm Hg]	92.2 ± 6.3 (87–96)	75.9 ± 4.7 (73–78)	<0.001
24 h ABPM MAP Z-score	1.52 ± 1.26 (0.71–2.04)	−1.05 ± 0.68 (−1.44–−0.78)	<0.001
PP 24 h	62.4 ± 7.2 (58.5–68)	50.9 ± 5.4 (47–55)	<0.001
HR [bpm]	77.0 ± 12.0 (69–87)	74.6 ± 9.4 (75–84)	0.338
24 h HR Z-score	−0.22 ± 1.39 (−1.14–0.68)	−0.66 ± 1.35 (−0.92–0.23)	0.176
SBPL/24 h (%)	57.8 ± 19.4 (41–74)	7.8 ± 6.2 (3–10)	<0.001
DBPL/24 h (%)	25.7 ± 19.4 (13–37)	3.9 ± 3.6 (1–6)	<0.001
24 h ABPM SBP SD	13.7 ± 2.5 (11.9–15.4)	12.6 ± 1.8 (11–14)	0.036
24 h ABPM DBP SD	11.3 ± 2.5 (9.5–12.8)	10.7 ± 2.1 (9.3–11.3)	0.225
24 h ABPM MAP SD	11.4 ± 2.6 (9.7–12.7)	10.0 ± 1.9 (8.7–10.9)	0.009
24 h ABPM HR SD	12.5 ± 3.4 (9.8–14.5)	12.0 ± 3.5 (9.1–14.6)	0.602
SBP DIP [%]	11.8 ± 5.5 (8.9–15.2)	12.9 ± 4.3 (10.5–16)	0.337
DBP DIP [%]	16.4 ± 8.6 (11–21.6)	19.6 ± 7 (15.7–24.4)	0.083
Morning BP surge [mm Hg]	10.9 ± 12 (4–18)	15.2 ± 8.1 (9–23)	0.066
24 h ABPM PP/SBP	0.46 ± 0.05 (0.43–0.48)	0.45 ± 0.03 (0.43–0.47)	0.128
24 h rate–pressure index [bpm x mm Hg]	10,352 ± 1584 (9205–11,228)	8466 ± 1203 (7810–9266)	<0.001
24 h weighted SBP variation	11.4 ± 1.8 (10.2–12.5)	10.3 ± 1.8 (9.1–11.8)	0.009
24 h weighted DBP variation	9.6 ± 1.7 (8.3–10.8)	8.7 ± 1.8 (7.6–10.3)	0.033
24 h weighted MAP variation	9.2 ± 1.6 (7.9–9.9)	8.0 ± 1.7 (6.8–9.8)	0.002
24 h coefficient of SBP variation [%]	10.2 ± 1.9 (8.9–11.5)	11.1 ± 1.6 (9.9–12.7)	0.012
24 h coefficient of DBP variation [%]	15.8 ± 3.5 (13.3–18.4)	17.1 ± 3.6 (14.8–18.1)	0.237
24 h coefficient of MAP variation [%]	12.4 ± 2.8 (10.3–14.1)	13.2 ± 2.4 (11.7–13.9)	0.134
AASI	0.39 ± 0.14 (0.32–0.50)	0.38 ± 0.11 (0.30–0.46)	0.709

SBP—systolic blood pressure; DBP—diastolic blood pressure; ABPM—ambulatory blood pressure monitoring; MAP—mean arterial pressure; PP—pulse pressure; HR—heart rate; SBPL—systolic blood pressure load; DBPL—diastolic blood pressure load; SD—standard deviation; DIP—blood pressure dipping; AASI—ambulatory arterial stiffness index.

**Table 3:** Diagnostic accuracy of markers of subclinical inflammation in predicting non-dipping status in children with primary hypertension.

Parameter	Area Under the Curve (95% CI)	*p*	Cut-Off Value	Sensitivity	Specificity	ACC
hsCRP [mg/L]	0.509 (0.344–0.675)	0.915	2.54	0.846	0.294	0.735
IL-18 [pg/mL]	0.520 (0.354–0.686)	0.810	7.7	0.294	0.821	0.661
Neutrophil count [×10^3^/µL]	0.574 (0.422–0.726)	0.341	3.48	0.765	0.487	0.571
Monocyte count [×10^3^/µL]	0.579 (0.416–0.742)	0.341	0.51	0.765	0.462	0.554
Lymphocyte count [×10^3^/µL]	0.656 (0.510–0.803)	0.037	2.59	0.588	0.744	0.696
Platelet count [×10^3^/µL]	0.659 (0.490–0.828)	0.065	320	0.353	0.974	0.786
Mean platelet volume (MPV) [fL]	0.534 (0.370–0.698)	0.685	10.6	0.590	0.647	0.607
Neutrophil-to-lymphocyte ratio (NLR)	0.502 (0.347–0.657)	0.977	2.54	0.179	1.000	0.429
Platelet-to-lymphocyte ratio (PLR)	0.525 (0.361–0.689)	0.766	122	0.538	0.647	0.571
Monocyte-to-lymphocyte ratio (MLR)	0.563 (0.401–0.725)	0.443	0.26	0.462	0.765	0.554
Monocyte-to-neutrophil ratio (MNR)	0.545 (0.382–0.708)	0.586	0.217	0.179	1.000	0.429
Platelet-to-MPV ratio (PMPVR) (10^12^/fL)	0.637 (0.470–0.803)	0.107	24.8	0.706	0.564	0.607

CI—confidence interval; ACC—accuracy; MPV—mean platelet volume; NLR—neutrophil-to-lymphocyte ratio; MNR—monocyte-to-neutrophil ratio.

**Table 4:** Multivariate analysis (general linear model) of determinants of blood pressure variability indices in patients with primary hypertension.

Blood Pressure Variability Index (Dependent Variable)	Predictor (Independent Variable)	Standardized Beta (95% CI)	*p*
24 h ABPM MAP SD	MNR	0.290 (0.029–0.551)	0.030
24 h rate–pressure index [bpm·mm Hg]	Age [years]	−0.336 (−0.572–−0.101)	0.006
	Monocytes [×10^3^/µL]	0.281 (0.041–0.521)	0.023
	MNR	−0.348 (−0.587–−0.108)	0.005
24 h weighted DBP variation	Age [years]	0.298 (0.001–0.596)	0.049
	hsCRP [mg/L]	0.310 (0.055–0.564)	0.018
	MNR	0.286 (0.032–0.540)	0.028
	Sex (M/F)	−0.364 (−0.663–−0.065)	0.018

CI—confidence interval; ABPM—ambulatory blood pressure monitoring; MAP—mean arterial pressure; SD—standard deviation; MNR—monocyte-to-neutrophil ratio; DBP—diastolic blood pressure; hsCRP—high-sensitivity C-reactive protein; M—male; F—female.

## Data Availability

Raw data are included within the Supplementary Materials files, Supplementary Table S1 (Table S1 patient_data.xlsx).

## Supplementary Materials

The following supporting information can be downloaded at: https://www.mdpi.com/article/10.3390/jcm14165737/s1. Table S1: Patient data (Table S1 patient_data.xlsx).
